# Supplementary material for: Functionally distinct and selectively phosphorylated GPCR subpopulations co-exist in a single cell
Source: Nat Commun. 2018 Mar 13;9:1050. doi: 10.1038/s41467-018-03459-7 (PMC5849717; doi:10.1038/s41467-018-03459-7)
Supplement: Supplementary file 1 — Supplementary Information [file 41467_2018_3459_MOESM1_ESM.pdf]

Supplementary Information for the Manuscript

Functionally distinct and selectively phosphorylated subpopulations of β2-adrenergic receptor co-exist in a single cell

Shen et al

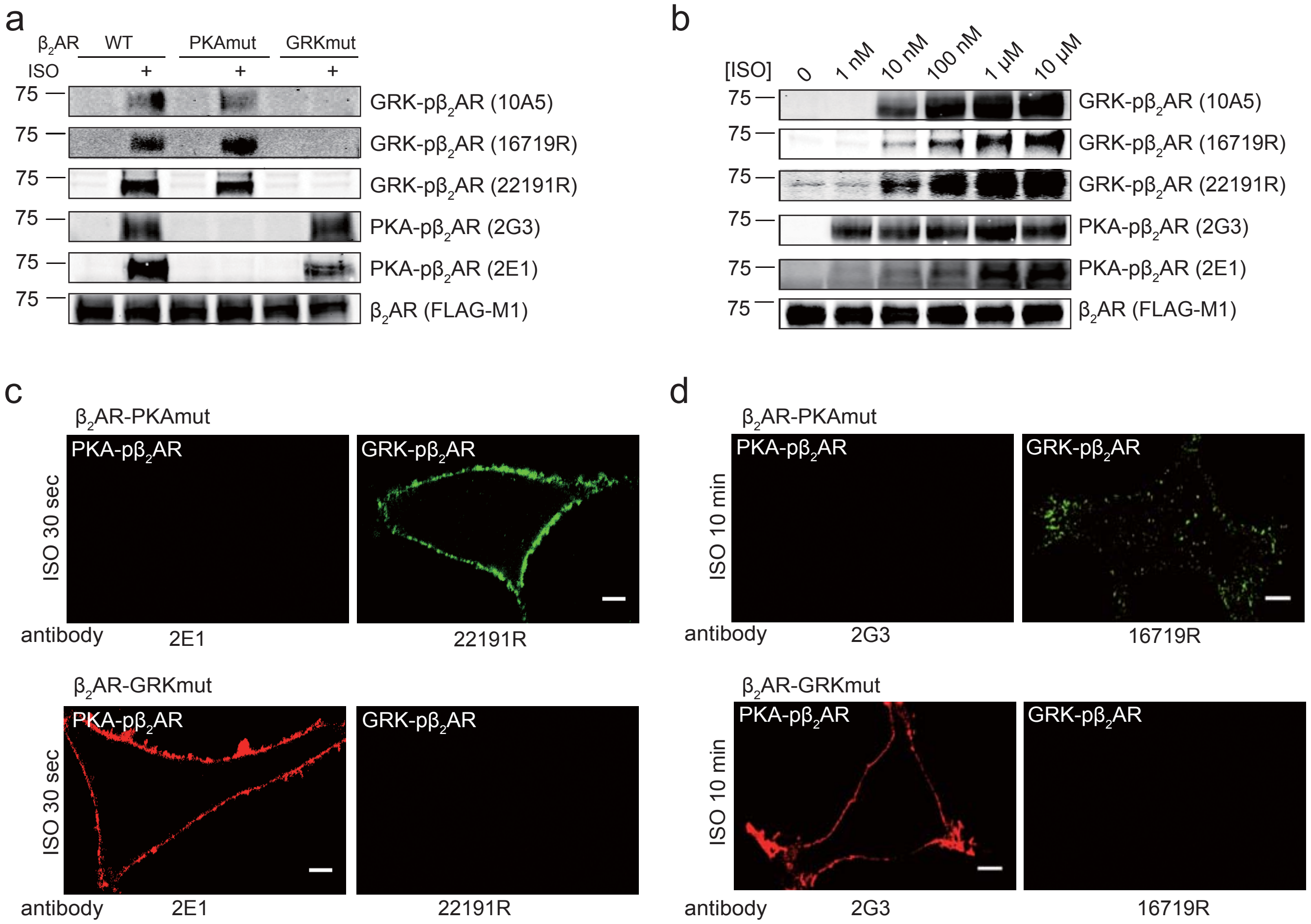

Supplementary Figure 1 Characterization of phospho-specific antibodies against β_2ARs by immunoblotting and immunofluorescence staining. HEK293 cells expressing FLAG-tagged WT β_2AR or mutant β_2AR lacking either PKA sites (PKAmut) or GRK sites (GRKmut) were stimulated with 1 μM ISO for 5 minutes or as indicated. **a,** Phospho-specific antibodies (monoclonal 10A5, polyclonal 16719R and 22191R against pS355/356; monoclonal 2G3 and 2E1 against pS261/262) selectively recognize phosphorylated β_2AR at PKA sites or GRK sites, respectively. Representative western blots of 4 independent experiments. Molecular weight markers (in kDa) are indicated on the left. **b,** Detection of phosphorylated β_2AR at PKA sites or GRK sites by phospho-specific antibodies after stimulation with different doses of ISO. **c** and **d,** Confocal imaging shows phosphorylated β_2AR labeled with phospho-specific antibodies in immunofluorescence staining as indicated. Scale bar, 5 μm. Representative of n = 6 cells per each condition from 3 independent experiments.

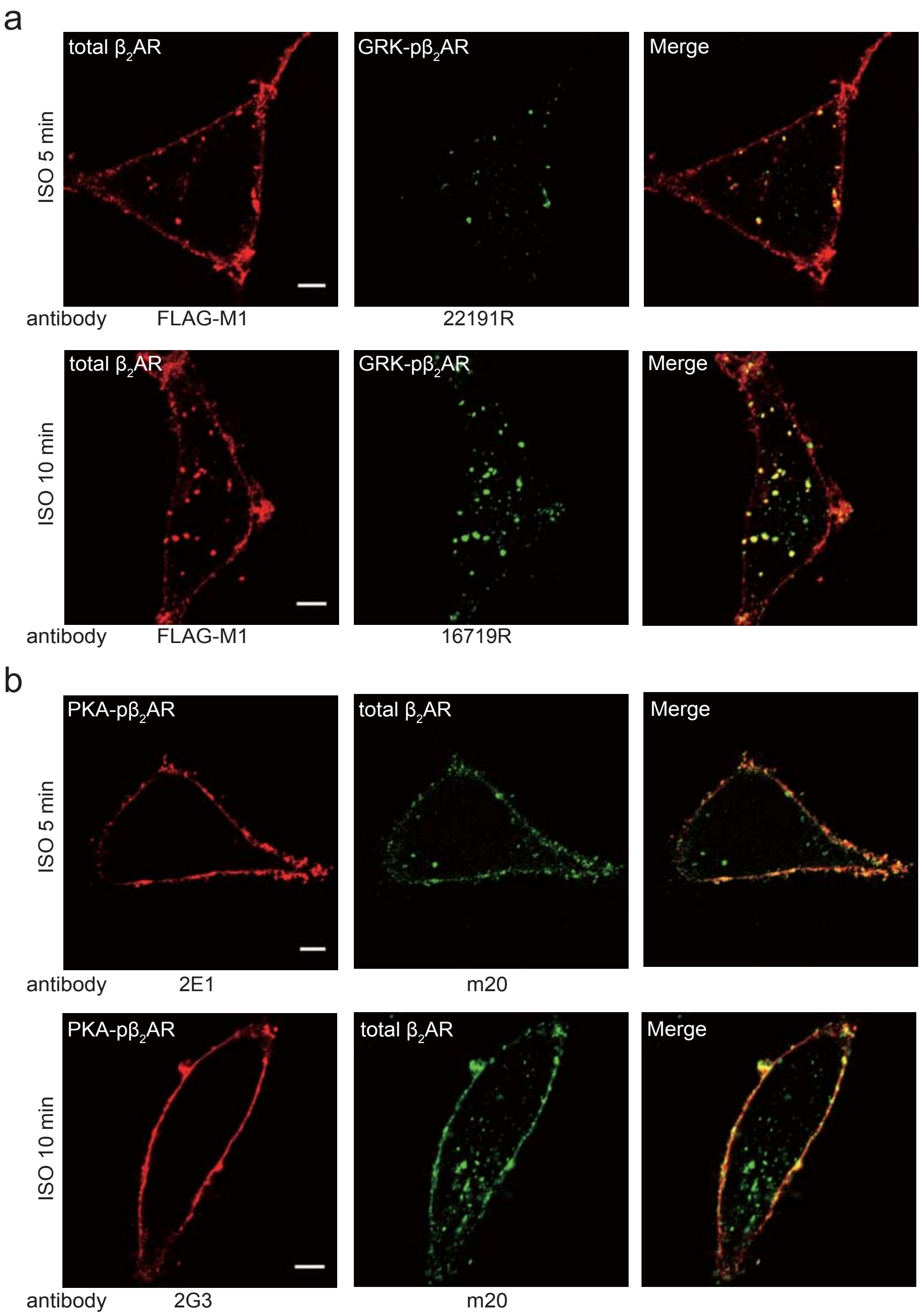

Supplementary Figure 2 PKA- and GRK-phosphorylated β_2ARs undergo distinct trafficking after prolonged stimulation with ISO. Confocal imaging shows subcellular location of total, PKA- and GRK-phosphorylated β_2AR in HEK293 cells expressing FLAG-β_2AR after stimulation with 1 μM ISO for indicated times. Phospho-specific antibodies against the PKA sites of pS261/262 and against the GRK sites of pS355/356 were used as indicated. Scale bar, 5 μm. Representative of n = 8 cells per each condition from 3 independent experiments.

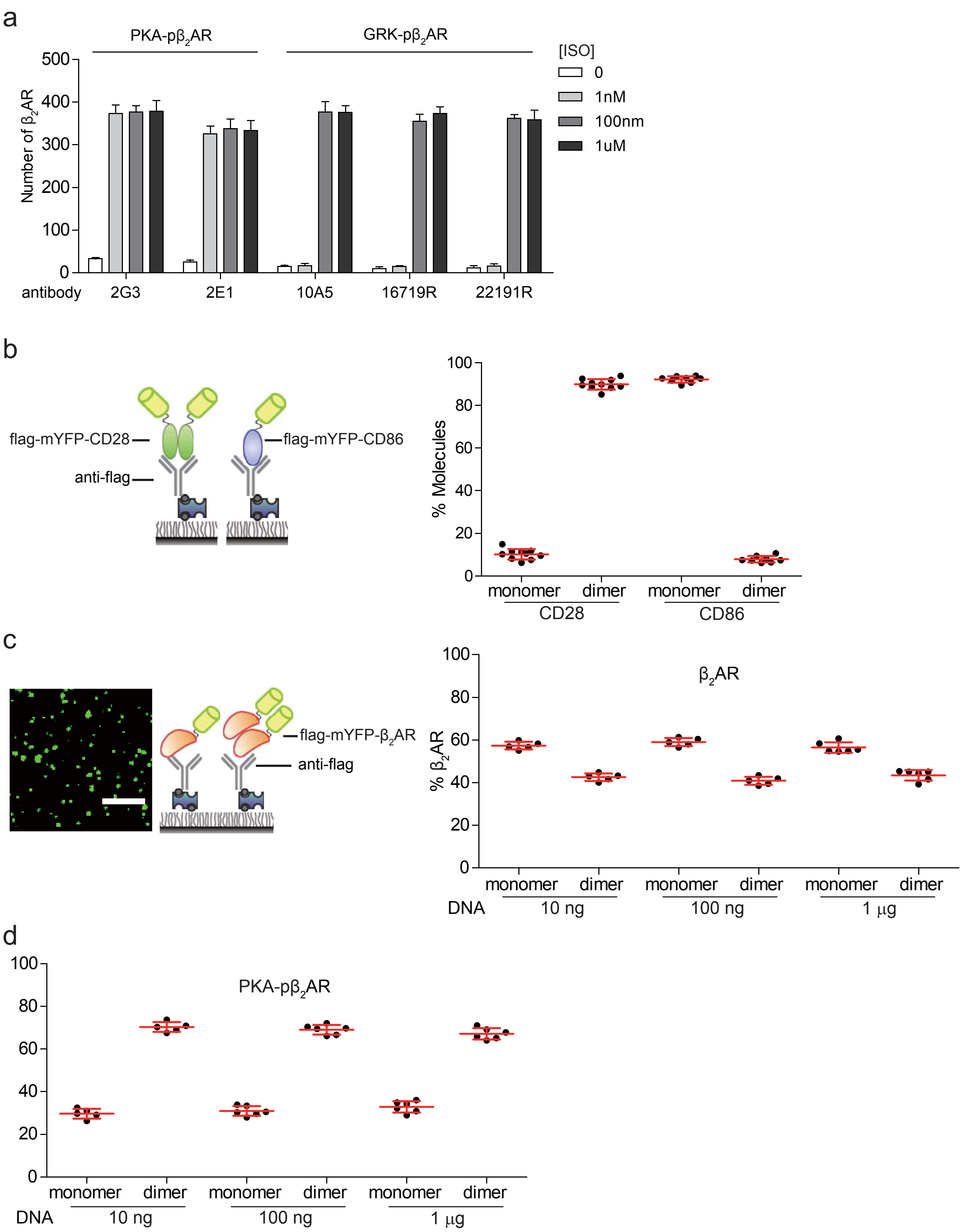

Supplementary Figure 3 Single molecule analysis of β$_2$AR stoichiometric composition. See next page for caption.

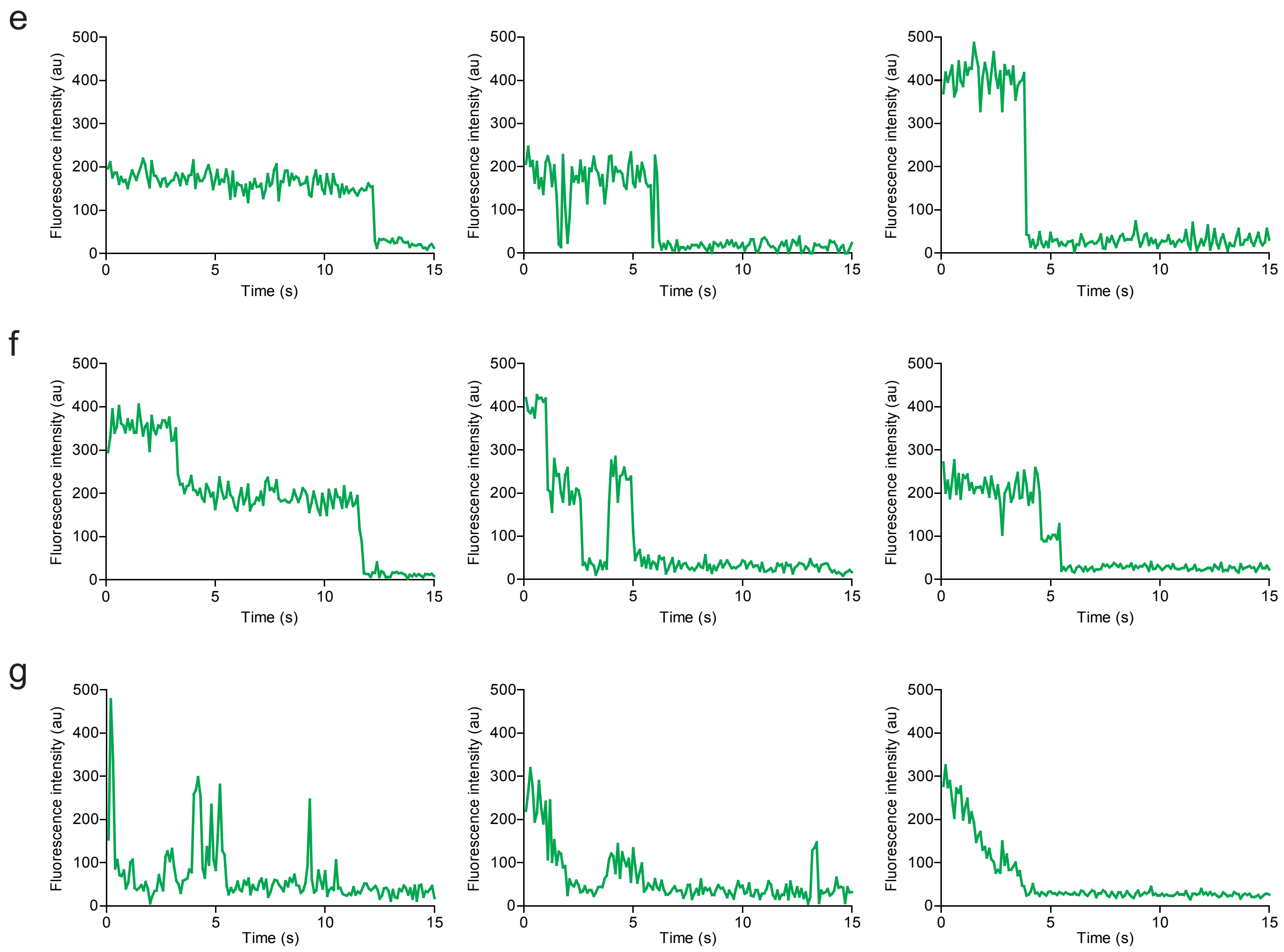

Supplementary Figure 3 Single molecule analysis of β_2AR stoichiometric composition. a, HEK293 cells expressing FLAG-mYFP-β_2AR were treated with ISO for 5 minutes at indicated concentrations. Numbers of β_2AR molecules pulled down by phospho-specific antibodies in SiMPull were quantified. n > 20. **b,** Schematic and representative SiMPull assay for analyzing stoichiometric composition of reference monomeric mYFP-CD86 and dimeric mYFP-CD28 proteins. CD86 displays more than 90% monomers whereas CD28 displays more than 90% dimers in SiMPull assay (n = 10 and 8 independent experiments, respectively). **c,** Schematic SiMPull assay for analyzing β_2AR stoichiometric composition. HEK293 cells were transfected with different amounts of FLAG-mYFP-β_2AR cDNA per 10^6 cells as indicated. Total β_2AR were pulled down with anti-flag in SiMPull before photobleaching assay. β_2AR composition was calculated based on fraction of photobleaching steps. Expression level of β_2AR did not affect the stoichiometric composition of total β_2AR (n = 5, 5 and 6 independent experiments, respectively). **d,** After stimulation with 1 μM ISO for 5 minutes, PKA-phosphorylated β_2AR were pulled down with pS261/262-specific antibody in SiMPull using same procedure as panel **c**. Expression level of β_2AR did not affect the stoichiometric composition of PKA-phosphorylated β_2AR (n = 5, 6 and 6 independent experiments, respectively). **e**-**g**, Samples of fluorescence traces depicting one-step (**e**) or two-steps (**f**) photobleaching of mYFP-β_2AR in SiMPull assay, which were used to calculate stoichiometric information of β_2AR. **g,** A fraction of molecules whose bleaching events could not be determined were excluded from stoichiometry analysis and scored under “rejected traces” in Supplementary Table 1. Error bars denote s.d. throughout the figure.

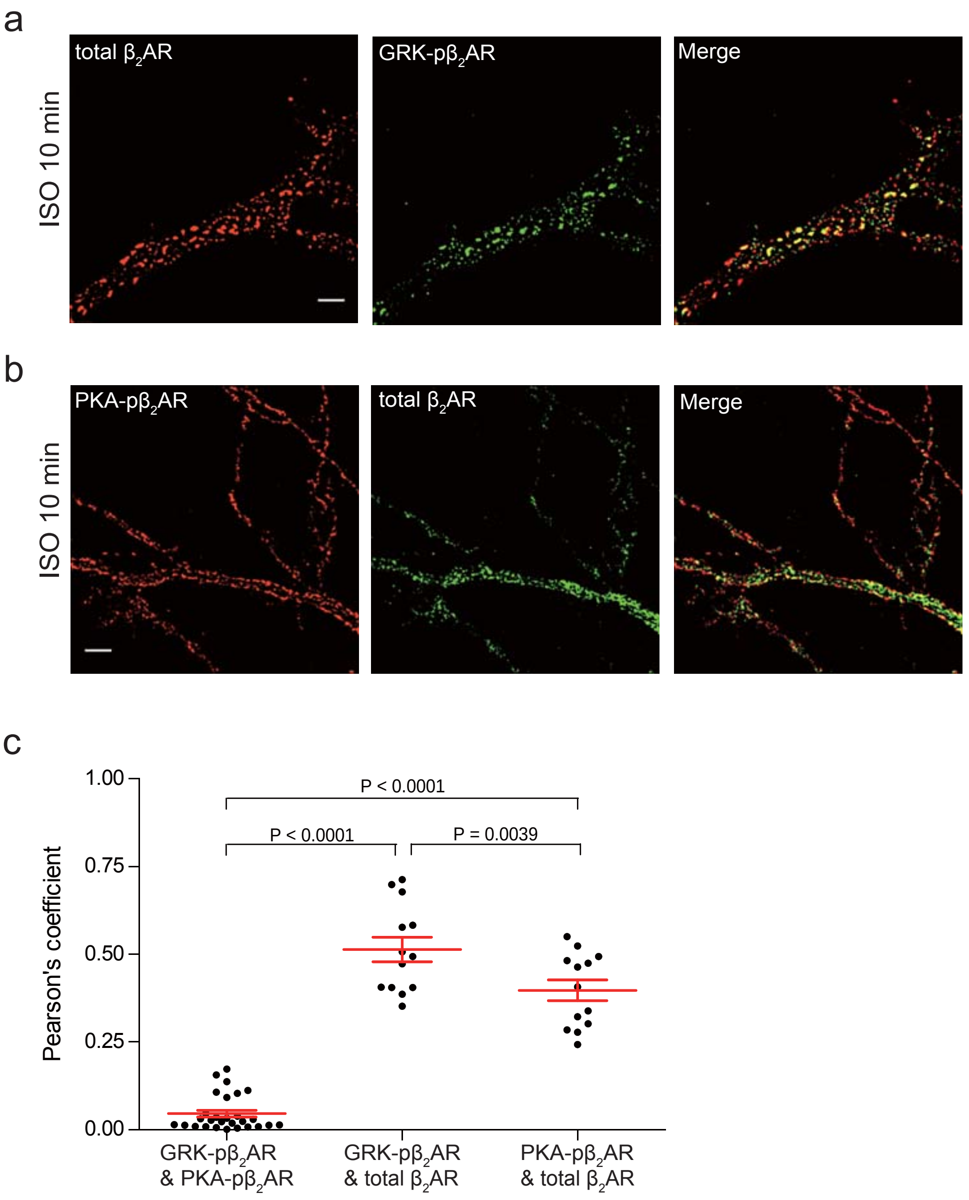

Supplementary Figure 4 Agonist induces PKA- and GRK-mediated phosphorylation of spatially segregated subpopulations of β_2AR in immature hippocampal neurons. a and **b,** SIM imaging of subcellular location of total, PKA- and GRK-phosphorylated FLAG-β_2AR in rat hippocampal neurons at 7 DIV after 10 minutes of stimulation with 1 µM ISO. Scale bar, 2 µm. Representative of n = 13 and 13 cells, respectively, 3 independent experiments. **c,** The overlap between two different stainings in panel **a** and **b** along with data shown in **Figure 4b** was evaluated by Pearson's correlation coefficient (n = 28, 13 and 13 cells, respectively). Error bars denote s.e.m., multiplicity adjusted P values are computed by one-way ANOVA followed by Tukey's test between indicated groups.

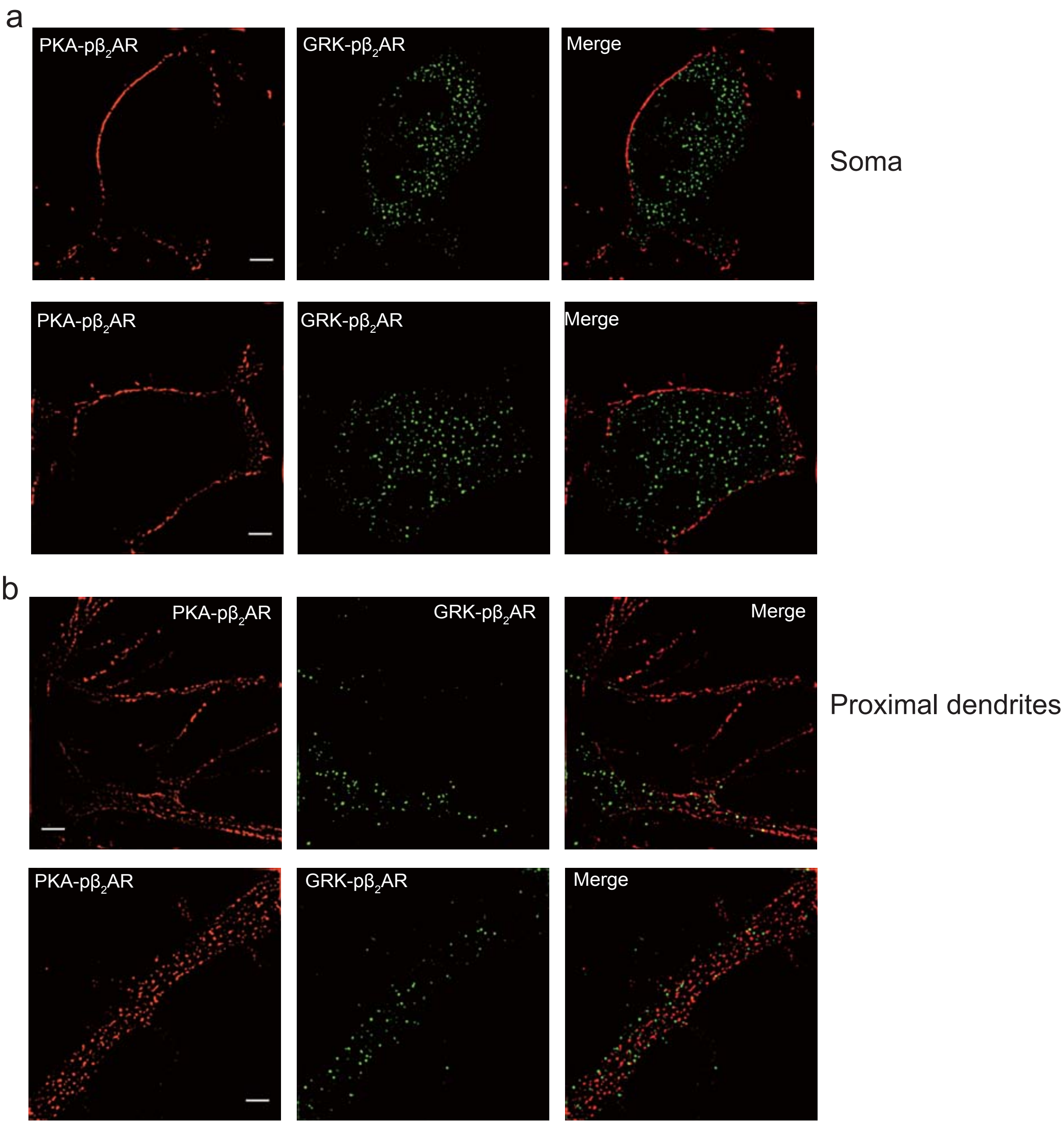

Supplementary Figure 5 Agonist induces PKA- and GRK-mediated phosphorylations of spatially segregated subpopulation of β_2AR in mature hippocampal neurons. See next page for caption.

c

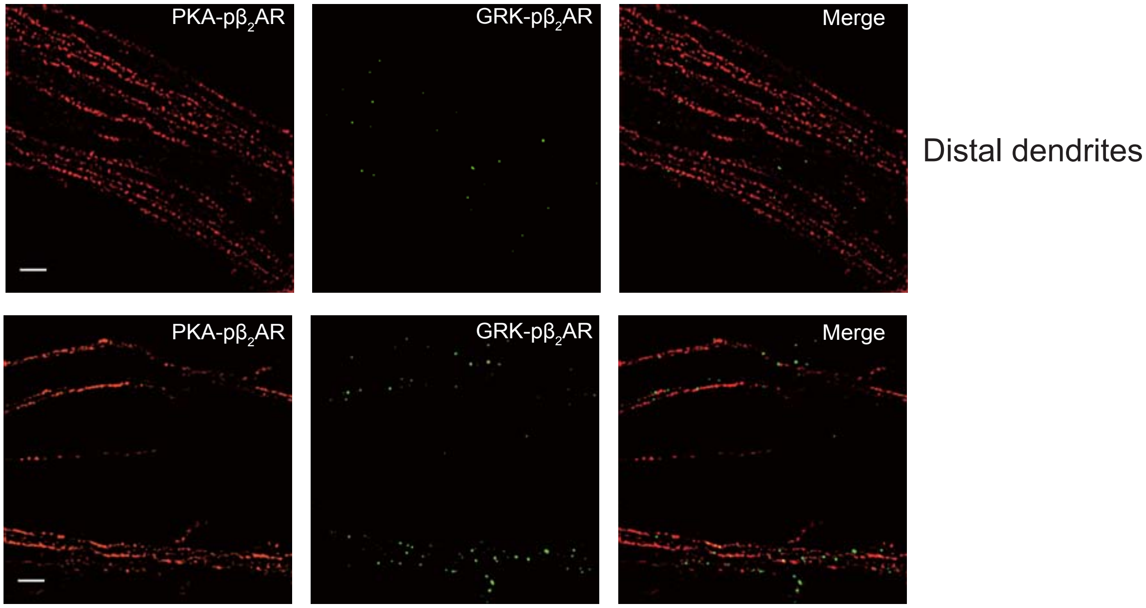

Supplementary Figure 5 Agonist induces PKA- and GRK-mediated phosphorylation of spatially segregated subpopulations of β_2AR in mature hippocampal neurons. Rat hippocampal neurons transfected with FLAG-β_2AR at 18-21 DIV were treated with 1 μM ISO for 5 minutes and probed with phospho-specific antibodies. SIM super-resolution imaging shows the distribution of PKA- and GRK-phosphorylated β_2ARs in soma (**a**), proximal dendrites (**b**), and distal dendrites (**c**) of mature hippocampal neurons. Scale bar, 2 μm.

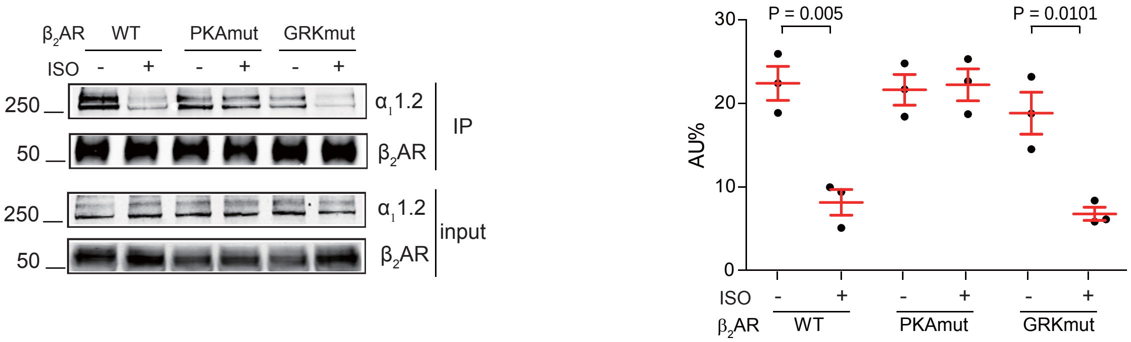

Supplementary Figure 6 PKA phosphorylation of β_2AR is necessary for agonist-induced dissociation of β_2AR from $\alpha_1$1.2. HEK293 cells expressing $\alpha_1$1.2 together with either WT or mutant FLAG-β_2ARs were either not stimulated (ND) or stimulated with 1 μM ISO for 5 minutes as indicated. The β_2AR was immunoprecipitated with anti-flag antibody. The coimmunoprecipitated $\alpha_1$1.2 was detected by Western blotting, and normalized to β_2AR (mean ± s.e.m.; exact P values are computed by two-tailed unpaired Student's t-test). Representative of 3 independent experiments. Molecular weight markers (in kDa) are indicated on the left.

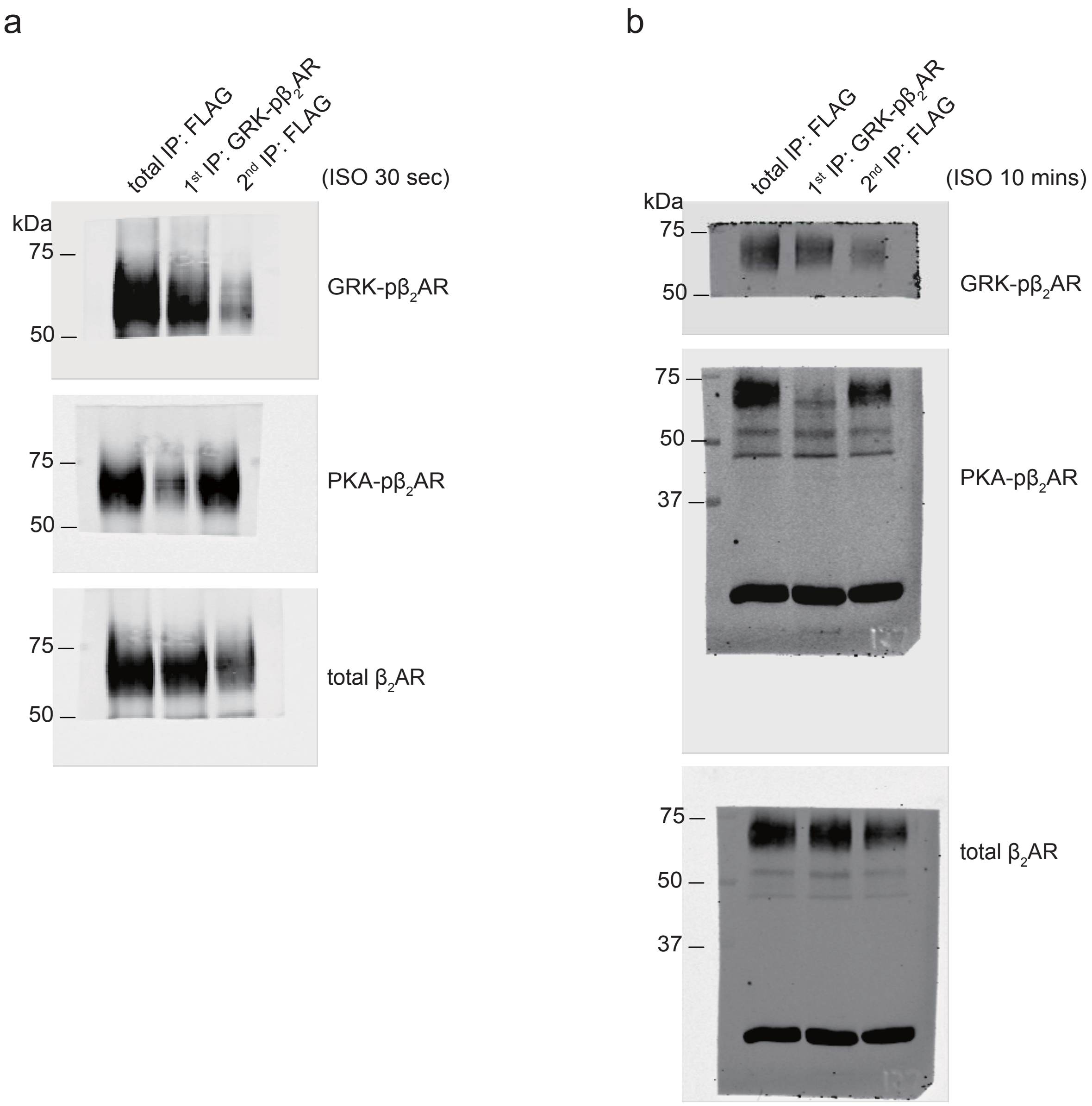

Supplementary Figure 7 Uncropped images of the Western blots shown in Figure 1e and Figure 2c. **a,** The gel images shown in Figure 1e is reproduced to show the entire blots. **b,** The gel images from Figure 2c is reproduced to show the entire blots. Molecular weight markers (in kDa) are indicated on the left.

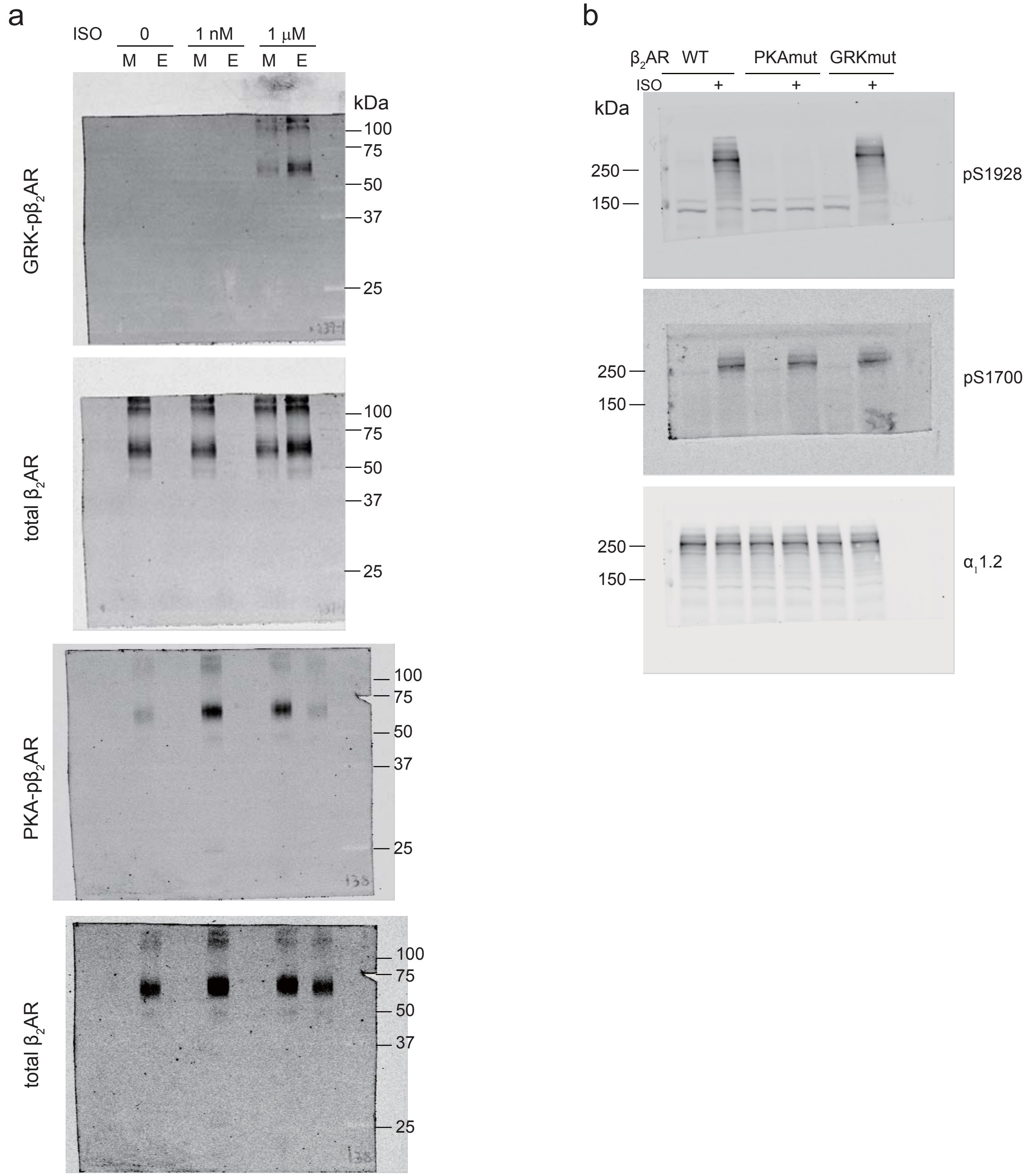

Supplementary Figure 8 Uncropped images of the Western blots shown in Figure 2d and Figure 7a. **a,** The gel images shown in Figure 2d is reproduced to show the entire blots with molecular weight markers (in kDa) indicated on the right. **b,** The gel images from Figure 7a is reproduced to show the entire blots with molecular weight markers (in kDa) indicated on the left.

Supplementary Table 1 Population distribution of observed fluorescence photobleaching steps and rejected traces

Anti body	Target protein	[ISO] (in nM)	Distribution of photobleaching steps*			
			1	2	>2	Rejected
FLAG	CD28	0	41.0% (378)	55.1% (508)	3.9% (36)	22.8% (272)
	CD86	0	93.3% (697)	5.7% (43)	1.0% (8)	17.9% (163)
FLAG	β_2AR	0	67.7% (1110)	28.8% (471)	3.5% (58)	25.8% (572)
		1	66.3% (1328)	30.4% (602)	3.3% (66)	25.4% (681)
		100	67.2% (817)	28.2% (342)	4.6% (56)	28.1% (478)
		1000	67.2% (1113)	28.8% (477)	4.0% (66)	26.7% (603)
FLAG	β_2AR (10ng)	0	66.8% (440)	28.9% (190)	4.3% (28)	24.6% (215)
	β_2AR (100ng)		68.7% (588)	27.8% (238)	3.5% (30)	21.8% (239)
	β_2AR (1ug)		66.4% (570)	29.8% (251)	3.8% (33)	20.9% (227)
2G3	β_2AR (10ng)	1000	50.3% (387)	44.9% (345)	4.8% (37)	26.5% (277)
	β_2AR (100ng)		51.3% (465)	44.2% (401)	4.5% (41)	27.3% (340)
	β_2AR (1ug)		52.1% (283)	43.1% (234)	4.8% (26)	26.1% (192)
2G3	β_2AR pS261/262	1	52.4% (715)	43.2% (590)	4.4% (60)	26.8% (493)
		100	50.2% (552)	45.3% (498)	4.5% (50)	26.5% (396)
		1000	51.2% (542)	44.3% (468)	4.5% (48)	26.5% (381)
2E1	β_2AR pS261/262	1000	54.8% (582)	42.7% (453)	2.5% (27)	25.1% (356)
10A5	β_2AR pS355/356	100	88.0% (874)	9.5% (94)	2.5% (25)	18.5% (225)
		1000	86.1% (827)	10.7% (103)	3.1% (30)	22.3% (276)
16719 R	β_2AR pS355/356	100	89.1% (890)	9.3% (93)	1.6% (16)	21.1% (267)
		1000	87.8% (936)	10.0% (107)	2.2% (23)	18.2% (237)
22191 R	β_2AR pS355/356	1000	89.1% (809)	9.4% (85)	1.5% (14)	19.1% (214)

* In parentheses: total number of molecules analyzed.
